# Interplay between surface chemistry and performance of rutile-type catalysts for halogen production†
†Electronic supplementary information (ESI) available: Additional computational details, additional HRTEM analysis, energy values, illustrations, and contour plots of the *ab initio* thermodynamics, transient responses of TAP experiments, reaction profile of the HX oxidation half cycle, and rate of halogen formation *versus* the halogen coverage. See DOI: 10.1039/c5sc04247j


**DOI:** 10.1039/c5sc04247j

**Published:** 2016-01-27

**Authors:** Maximilian Moser, Vladimir Paunović, Zhen Guo, László Szentmiklósi, Miguel G. Hevia, Michael Higham, Núria López, Detre Teschner, Javier Pérez-Ramírez

**Affiliations:** a Institute for Chemical and Bioengineering , Department of Chemistry and Applied Biosciences , ETH Zurich , Vladimir-Prelog-Weg 1 , 8093 Zurich , Switzerland . Email: jpr@chem.ethz.ch; b Centre for Energy Research , Hungarian Academy of Sciences , Konkoly-Thege Miklós Street 29-33 , 1121 Budapest , Hungary; c Institute of Chemical Research of Catalonia , ICIQ , Barcelona Institute of Science and Technology , BIST , Av. Països Catalans 16 , 43007 Tarragona , Spain . Email: nlopez@iciq.es; d Fritz-Haber-Institute of the Max Planck Society , Faradayweg 4-6 , 14195 Berlin , Germany . Email: teschner@fhi-berlin.mpg.de

## Abstract

Catalytic HBr oxidation is an integral step in the bromine-mediated functionalisation of alkanes to valuable chemicals. This study establishes the relationships between the mechanism of HBr oxidation over rutile-type oxides (RuO_2_, IrO_2_, TiO_2_) and their apparent catalytic performance. Comparison with the well-studied HCl oxidation revealed distinct differences in surface chemistry between HBr and HCl oxidation that impact the stability and activity of the catalysts. The kinetic fingerprints of both oxidation reactions over the three rutile-type oxides investigated are compared using temporal analysis of products, which substantiates the energy profiles derived from density functional theory. The quantitative determination of the halogen uptake under *operando* conditions using prompt gamma activation analysis demonstrates that RuO_2_ suffers from extensive subsurface bromination upon contact with hydrogen bromide, particularly at low temperature and low O_2_ : HBr ratios, which negatively affects the stability of the catalyst. TiO_2_ exhibits intrinsically low halogen coverage (30–50%) under all the conditions investigated, due to its unique defect-driven mechanism that renders it active and stable for Br_2_ production. On the contrary, for HCl oxidation TiO_2_ is inactive, and the chlorination of the highly active RuO_2_ is limited to the surface. Differences in the extent of surface halogenation of the materials were also confirmed by high-resolution transmission electron microscopy and explained by the DFT calculations. These insights into the molecular-level processes taking place under working conditions pave the way for the design of the next generation catalysts for bromine production.

## Abstract 2

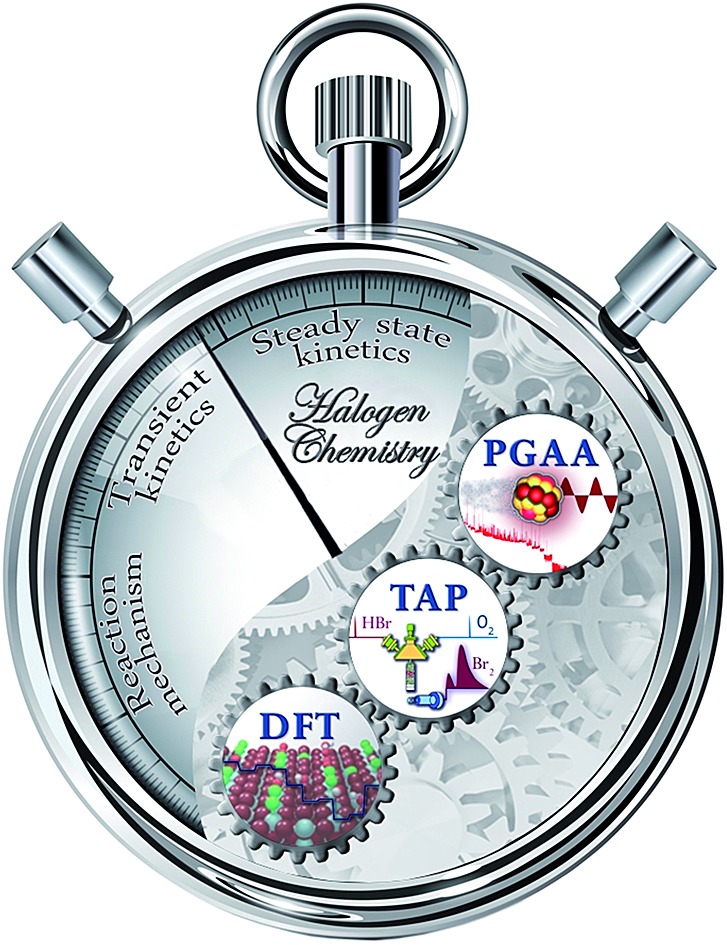
DFT, TAP, and *operando* PGAA studies demonstrate that the complex and dynamic surface chemistry of hydrogen halide oxidation on rutile-type catalysts determines the activity and stability patterns for halogen production.

## Introduction

The surface chemistry of metal oxides that exhibit a rutile structure is rich and complex.^1^ Their dense lattice structure renders these materials stable against harsh reaction conditions and makes them an attractive option for applications in heterogeneous, electro-, and photo-catalysis.^1^,^2^ Recently, the implementation of RuO_2_-based catalysts for the recovery of chlorine *via* the oxidation of HCl, a copious by-product of polyurethane and polycarbonate production, stimulated multiple studies employing interdisciplinary methods.^1^,^3^ It was demonstrated that the stability of RuO_2_ against bulk chlorination originates from a self-limiting mechanism that confines the chlorination to the surface, while its low temperature activity is linked to the small energetic barrier for the evolution of chlorine.^4^*Operando* studies on RuO_2_ showed that the activity is related to the extent of surface chlorination and can be controlled through variation of temperature and oxygen partial pressure.^5^

Rutile-type catalysts were also studied for the gas-phase oxidation of HBr to Br_2_, a crucial step in closing the bromine cycle in emerging bromine-mediated alkane functionalisation processes.^6^ It was found that RuO_2_- and IrO_2_-based systems catalyse HBr oxidation at much lower temperatures than HCl oxidation, due to the lower energy barrier of bromine evolution compared to that of chlorine evolution.6*d* Further studies resulted in the discovery of TiO_2_ as an active catalyst for HBr oxidation, despite its inertness in HCl oxidation.^7^ Molecular modelling studies proposed that a self-doping mechanism with bromine enables the adsorption and activation of molecular oxygen on TiO_2_.^7^ These results underlined that the mechanism of HBr oxidation is more multifarious than that of HCl oxidation on rutile surfaces. However, there is a lack of systematic studies that enable one to rationalise the relationship between the state of the surface and the apparent catalytic performance of different rutile-type catalysts in HBr oxidation. A comprehensive mechanistic picture can be developed through a multidisciplinary approach combining theoretical calculations with *ex situ* and *operando* methods.

Herein, we undertake a detailed study of the surface chemistry of rutile-type catalysts, *i.e.* RuO_2_, IrO_2_, and TiO_2_, in HBr oxidation, including a direct comparison to HCl oxidation. Advanced characterisation techniques provide an in-depth perspective on the catalytic hydrogen halide oxidation that goes beyond the experimental limitations of previous studies on HBr oxidation. Analysis of microscopic imaging and energy profiles calculated from Density Functional Theory rationalise the interactions of the gaseous environment with the catalysts. The dynamics of product formation are deduced from temporal analysis of products. These are combined with *operando* prompt gamma activation analysis experiments under steady-state conditions, enabling determination of the halogen surface coverage. Merging these results, we discuss the impact of surface halogenation by hydrogen halides on the structural stability of the rutile materials and the mechanistic fingerprints of hydrogen halide oxidation over these catalysts.

## Experimental

### Catalyst preparation

The as-received RuO_2_ (11 m^2^ g^–1^, Aldrich, 99.5%) and TiO_2_ (20 m^2^ g^–1^, Aldrich, rutile nanopowder, 99.5%) powders were calcined at 723 and 823 K, respectively, using a ramp of 5 K min^–1^ and a holding time of 5 h. IrO_2_ (9 m^2^ g^–1^) was prepared by calcination of anhydrous IrCl_3_ (ABCR, 99.9%) at 823 K for 5 h using a heating rate of 5 K min^–1^.

### High-resolution transmission microscopy

High-resolution transmission electron microscopy (HRTEM) was undertaken on either a FEI Tecnai F30 microscope operated at 300 kV or a FEI Talos microscope operated at 200 kV. Elemental mapping using energy-dispersive X-ray spectroscopy (EDXS) was carried out in STEM-mode with 4 X-ray detectors (Bruker) attached to the Talos microscope. Prior to microscopic analysis, the solids were dispersed in ethanol; a few droplets of the suspension were poured onto a holey carbon-coated copper grid and dried completely.

### Computational details

Density functional theory (DFT), as implemented in the Vienna *ab initio* simulation package (VASP) version 5.3.3, was applied to slabs representing the rutile (110) facet, for both RuO_2_ and TiO_2_.^8^ For all rutile-type catalysts, the (110) surface represents the largest contribution to the surface in the Wulff construction of the nanoparticles. For example, RuO_2_ exhibits 43% of the (110) facet and a complementary 42% of the (101) facet.5*b* For calculations involving RuO_2_(110), a *p*(2 × 1) supercell was employed, with a *k*-point sampling of 4 × 4 × 1, using the scheme of Monkhorst and Pack.^9^ For calculations involving TiO_2_(110), the chosen slab corresponds to a *p*(2 × 2) supercell, with a *k*-point sampling of 4 × 2 × 1. In both cases, slabs consisting of five layers were interleaved by a 15 Å vacuum. The top two layers of the slabs were optimised, while the remaining bottom three were fixed. Forces were converged to within 0.015 eV Å^–1^. In order to eliminate the spurious electrostatic interactions associated with asymmetric relaxation of the slab, a dipole correction was applied to the vacuum. The exchange-correlation functional PBE,^10^ as well as PBE+U^11^ for TiO_2_, was employed, with *U*_eff_ = 4.5 eV for Ti. Inner electrons were replaced with PAW,^12^ and the valance states were expanded in plane-waves with a cut-off energy of 450 eV. Spin-polarised calculations were performed as necessary. Transition states were identified by CI-NEB.^13^*Ab initio* thermodynamics calculations were performed using lowest energy surface configurations to determine the surface composition under typical experimental conditions as a function of the partial pressures, *p*(HX), *p*(H_2_O), and *p*(X_2_); (X = Cl, Br). The experimental gas-phase thermodynamic data from the NIST reference tables were utilised in conjunction with the DFT results for the relevant molecular species (*viz.* HCl, HBr, Cl_2_, Br_2_, and H_2_O).^14^ The simulations were conducted under various partial pressures and temperatures for RuO_2_ and TiO_2_ with different degrees of halogenation. Additional computational details with regard to halogen substitution in the rutile structures are provided in the ESI.† Note that the results of the *ab initio* thermodynamics indicate the most stable configuration of the material as a function of external temperatures and pressures. Thus, the lowest energy halide distributions are identified, but not the process (kinetics or mechanism) of how subsurface halide uptake takes place.

### Temporal analysis of products (TAP)

Transient mechanistic studies of HBr and HCl oxidation over RuO_2_, IrO_2_, and TiO_2_ were carried out in a TAP-2 reactor.^15^ The samples (10 mg, particle size = 0.2–0.4 mm) were loaded in the isothermal (central) zone of a stainless steel micro-reactor (4.6 mm internal diameter) between two layers of quartz particles of size 0.125–0.2 mm. The thickness of the catalyst zone (1–2 mm) was very small compared with the overall bed length (37 mm). This configuration, referred to as a thin-zone reactor, is characterised by negligible gas concentration gradients across the catalyst bed.15*b* The samples were pre-treated in a flow of 20 cm^3^ STP min^–1^ O_2_ at 623 K and 1 bar for 1 h, followed by evacuation of the reactor to 10^–10^ bar. The following pulse experiments were carried out in high vacuum at 523, 573, and 623 K for HBr and at 623 K for HCl oxidation, using a pulse size of *ca.* 10^16^ molecules: (a) individual pulsing of either O_2_ : He = 8 : 1 or HX : Ar = 16 : 1 (X = Cl, Br); (b) simultaneous pulsing of O_2_ : He = 8 : 1 and HX : Ar = 16 : 1; and (c) pump-probe pulsing of O_2_ : He = 8 : 1 and HX : Ar = 4 : 1. The latter is also referred to as sequential pulsing, where the pulses of the two gas mixtures are separated by a time delay (Δ*t*). The pulses were separated by 0.25 and 1 s for O_2_ : He (pump pulse) and HX : Ar (probe pulse). In the case of HX : Ar (pump pulse) and O_2_ : He (probe pulse), the delays were 2 and 8 s. The pump and probe pulses were repeated cyclically, keeping a constant time interval of 8 s between the probe pulse in one cycle and the pump pulse in the following cycle in all cases.

In the experiments, Ar (Linde, purity 5.0), He (Air Products, purity 5.2), O_2_ (Air Products, purity 5.2), HBr (Linde, purity 4.5), and HCl (Linde, purity 2.8) were used. A quadrupole mass spectrometer (RGA 300, Stanford Research Systems) monitored the transient responses at the reactor outlet of the following atomic mass units (AMUs): 160 (Br_2_), 80 (HBr), 70 (Cl_2_), 40 (Ar), 36 (HCl), 32 (O_2_), 18 (H_2_O), and 4 (He). The responses displayed correspond to an average of 20 pulses per AMU to improve the signal-to-noise ratio. Prior to that, it was verified that the responses were stable; that is, with invariable intensity and shape during at least 40 consecutive pulses.

### *Operando* prompt gamma activation analysis

The Br and Cl uptake on the rutile-type catalysts during HBr and HCl oxidation was determined using *operando* prompt gamma activation analysis (PGAA) using the cold neutron beam at the Budapest Neutron Centre. The technique was successfully used to study the surface chlorination of Deacon catalysts^5^,^16^ and is based on neutron capture by the atomic cores in the sample and the subsequent detection of element-specific gamma rays emitted during de-excitation of the nuclei. The gas-phase oxidation of hydrogen halides was studied in a quartz fixed-bed micro-reactor (8 mm internal diameter) at ambient pressure and in the temperature range of 413–713 K. The reactor was loaded with 0.5 g of RuO_2_ or 0.23 g of TiO_2_ catalyst (particle size = 0.4–0.6 mm) and was placed inside a furnace in the path of the neutron beam. A total volumetric flow of 250 cm^3^ STP min^–1^ consisting of 10 vol% HBr or HCl (Air Liquide, anhydrous) and 0–90 vol% O_2_ (Linde, purity 4.5) balanced in N_2_ (Linde, purity 5.0) was continuously fed into the reactor. Furthermore, 0–4 vol% Br_2_ (Acros, 99.8%) was added to the gas feed by passing part of the N_2_ flow through a saturator filled with liquid Br_2_ under STP conditions. The halogen formation was quantified by iodometric titration using a Mettler Toledo G20 Compact Titrator, as detailed elsewhere.4*d* The rate of halogen formation was expressed as moles of halogen produced per gram of catalyst and unit of time. The cold neutrons were guided to the experimental station by curved supermirror neutron guides. A Compton-suppressed high-purity germanium (HPGe) crystal detects the prompt gamma photons emitted by the catalyst bed. Br, Cl, Ru, and Ti were determined from the characteristic peak areas. The surface coverage was calculated as the amount of halogen per surface site, taking into account the surface area of the catalysts and the density of sites on the (110) and (101) facets, weighted by their relative occurrence.5a,^17^ The gas-phase signals of HBr, Br_2_, HCl, and Cl_2_ were subtracted; thus, all reported values correspond to the solid catalyst only.

## Results and discussion

### Halogenation of rutile structures

**Fig. 1:**
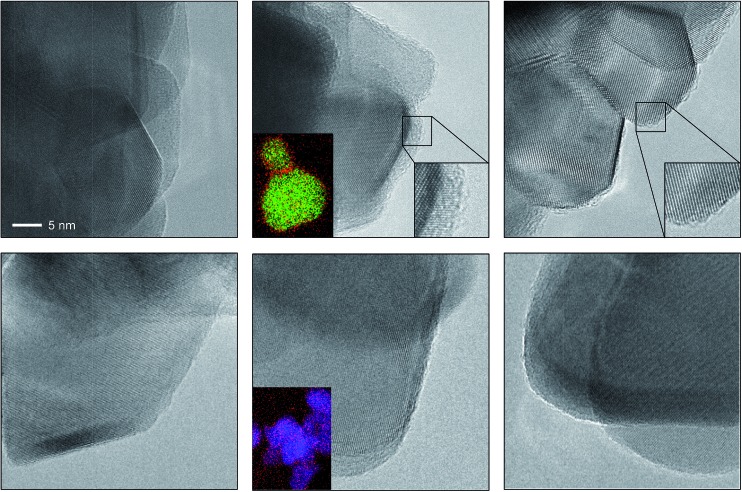
HRTEM images of RuO_2_ (top) and TiO_2_ (bottom) showing the as-prepared materials (left), and the materials after treatment in HBr at 393 K (centre) and HCl at 543 K (right). The scale bar in the top left image applies to all images. The bottom left insets in the centre micrographs depict the averaged elemental mapping of the RuO_2_ and TiO_2_ particles using EDXS, with Ru shown in green, Ti shown in blue and Br shown in red. The bottom right insets in the top centre and right micrographs show magnified images of the amorphised particle edges. Additional micrographs of RuO_2_ and TiO_2_ (Fig. S1 and S2†) and structural models (Fig. S4 and S5†) of brominated RuO_2_ are provided in the ESI.†

The depicted nano-particles of RuO_2_ and TiO_2_ are *ca.* 20 nm and 50 nm in size, respectively, and exhibit sharp edges with clearly visible fringes, providing a high degree of crystallinity as shown by HRTEM (Fig. 1). After treatment in both HBr and HCl for 3 h (*i.e.* O_2_ : HX = 0), both RuO_2_ and TiO_2_ particles retained their size, while the RuO_2_ surfaces exhibited significant amorphisation compared to TiO_2_. The evaluation of the RuO_2_ crystals showed that the amorphised layer is thicker after treatment in HBr compared to HCl (Fig. 1 and S1†). Analysis of the HBr-treated samples with EDXS corroborated this observation, detecting a significant concentration of bromine along the particle edges (Fig. 1, middle). The TiO_2_ surfaces were virtually unaffected by the HBr and HCl treatment. The EDXS mapping of TiO_2_ did not detect significant traces of chlorine, and only small amounts of bromine were observed (Fig. 1 and S2†). These results are further supported by the measured halogen uptake under the reaction conditions, which indicates a four times higher bromine uptake on RuO_2_ compared to chlorine and comparably low halogen uptake on TiO_2_ (*vide infra*).

In previous studies, it has been observed that rutile-type catalysts used in heterogeneous catalysis are affected by the chemical environment in which the reaction takes place. Theoretical investigations of the active phase can be conducted at different levels of complexity. For instance, bulk stability against complete halogenation (*i.e.* where bulk MO_2_ is replaced by MX_*n*_) and the possibility of rutile degradation *via* the formation of volatile species (TiCl_4_ formation would be an example) could also be considered. However, to address the formation of mixed phases it is more illustrative to conduct simulations of the substitution of surface and subsurface oxygen atoms by halogen atoms in the lattice of RuO_2_. The theoretical replacement energies reported were calculated according to the equation RuO_2_ + 2*n*HX → RuO_2–*n*_@X_*n*_ + *n*H_2_O + *n*/2X_2_, where *n* is the number of oxygen atoms replaced and X refers to either Br or Cl. Lattice oxygen enters the gas phase in the form of water. The energies for RuO_2_ bromination are most exothermic for configurations corresponding to the substitution of two O atoms (Δ*E*_r_ = –1.97 eV, Fig. S3, 2A†), and remain significantly exothermic for up to four Br atoms (Δ*E*_r_ > –1 eV, Fig. S3, 4B†). Upon addition of the first subsurface Br atom, the bromination becomes less exothermic (Δ*E*_r_ = –0.50 eV, Fig. S3, 5C†), and eventually it becomes slightly endothermic after the addition of a further Br atom (Δ*E*_r_ = 0.30 eV, Fig. S3, 6H†). Notably, the incorporation of four or more Br atoms into the lattice already leads to a considerable degree of structural rearrangement (Fig. S4, 4B and F†). The surface Br atoms relax such that they form a hexagonal arrangement (Fig. S5†). The high Br density of these structures represents a change in the geometry that would appear as amorphisation in the experiments (Fig. 1, and S1†). Furthermore, considering factors unaccounted for by the above methodology, such as configurational entropy or other surface orientations, extensive subsurface bromination is well within the bounds of possibility.4*c* The present calculations only aim at establishing the lowest energy configurations and do not provide any indication as to how the bromination occurs. For RuO_2_ chlorination, the process is only exothermic for up to two Cl atoms incorporated into the surface (Δ*E*_r_ = –0.93 eV, Fig. S3, 2A†), with subsequent Cl addition being endothermic (Fig. S3b, 3A†). While incorporation of up to four Cl atoms is moderately endothermic (Δ*E*_r_ = 0.71 eV, Fig. S3, 4B†), subsequent chlorination of the subsurface layers is considerably more so, reaching Δ*E*_r_ = 1.81 eV and 3.21 eV for five and six Cl atoms, respectively (Fig. S3, 5C† and 6H†).

**Fig. 2:**
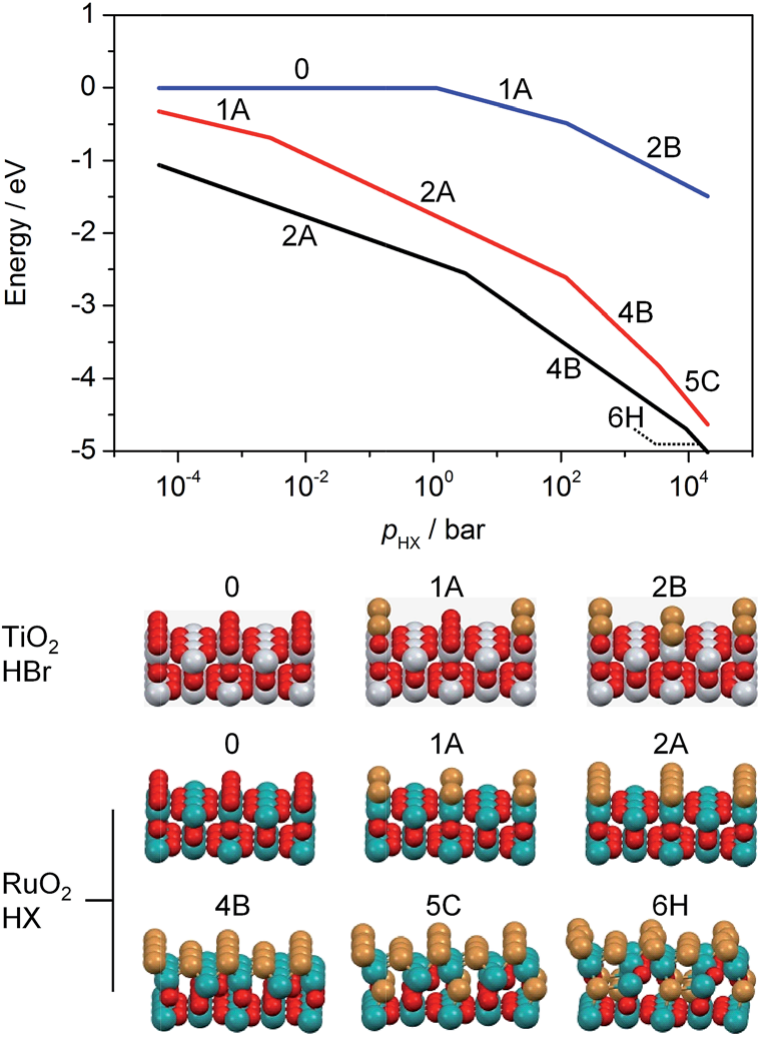
Free energy of substitution *versus* the partial pressure of the hydrogen halide for the bromination of TiO_2_ (blue) and RuO_2_ (black), as well as for the chlorination of RuO_2_ (red). The partial pressures of X_2_ and H_2_O were fixed at 10^–4^ bar. The temperature was set to 573 K (TiO_2_) and 393 K (RuO_2_) for bromination and 523 K (RuO_2_) for chlorination. The labels in the plot correspond to the structures shown below, which exhibit the lowest free energy under the simulated conditions. These depict the substitution of Cl or Br atoms into the surface and subsurface layers. Colour code: Ti (grey), Ru (green), O (red), and Cl or Br (brown).

*Ab initio* thermodynamic studies were performed to identify which halogenated structures would be stable under the treatment conditions, *i.e.* variable HX and low product partial pressures (Fig. 2).‡
‡The *ab initio* thermodynamics should be interpreted with caution as a number of approximations (*i.e.* configurational entropies are missing) have been implicitly included, and thus this model only serves for qualitative purposes. The results show that the surface composition consists of between two and four Br atoms or two Cl atoms per (2 × 1) supercell between *p*(HX) = 10^–2^ and 100 bar.§
§The preference for bromination can be seen from the fact that the transition between the 2A and 4B configurations occurs at lower pressures than for Cl. Higher *p*(HX) favours more extensively halogenated structures, while the free energy for substitution of Br atoms remains more exothermic than that for Cl atoms for all partial pressures in the range considered. This indicates that bromination is stronger under comparable conditions, as observed in the microscopic analysis. The impact of *p*(H_2_O) and *p*(X_2_) on the RuO_2_ surface composition is less pronounced (Fig. S6 and S7†), with the surface structure showing considerably less variation over the same pressure range as compared to *p*(HX).

The calculations for TiO_2_, on the other hand, show that Br uptake is endothermic for incorporation of both one and two Br atoms, with Δ*E*_r_ = 0.43 eV and 1.33 eV, respectively (Fig. S8, 1A and 2B†). The lowest energy configuration for two Br atoms had them both occupying bridging sites in diagonal fashion, as this would result in minimal repulsion between them (Fig. S8a, 2B†). Our *ab initio* thermodynamics suggest that under typical experimental conditions, *i.e.* low *p*(H_2_O) and *p*(Br_2_), as well as high *p*(HBr), the Br uptake is limited, with a maximum of one Br atom per (2 × 2) surface cell. Furthermore, the free energy of substitution of a Br atom into TiO_2_ is much lower than those of RuO_2_ chlorination and bromination (Fig. 2). The product partial pressures have a very limited impact on the surface bromination, thus indicating that the HX partial pressure is the dominant factor in determining the halogenation of rutile-type structures.

### Mechanistic studies

**Fig. 3:**
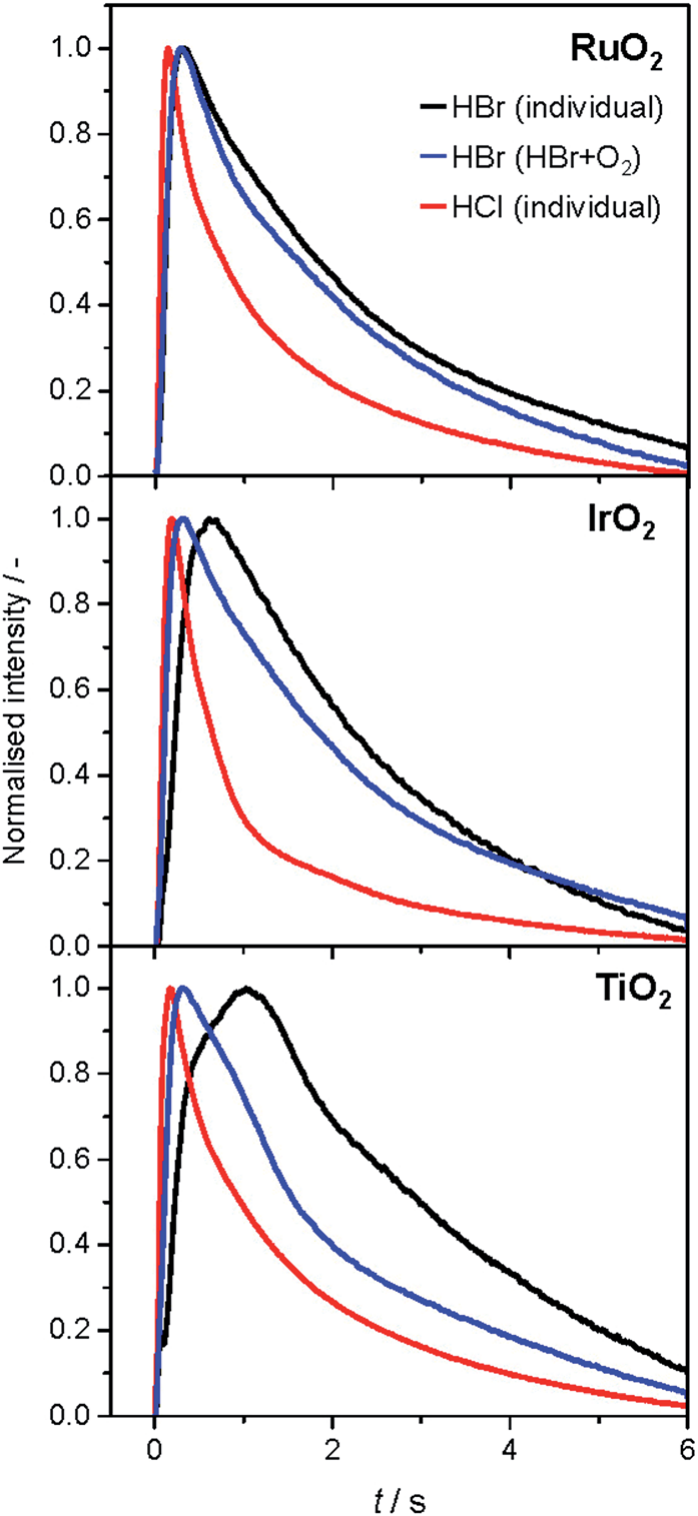
Normalised transient responses of HBr and HCl after individual (HBr, black; HCl, red), and simultaneous (HBr + O_2_, blue) pulse experiments at 623 K.

The analysis of the reaction mechanism of HBr and HCl oxidation was conducted by combining pulse experiments in a TAP-2 reactor with DFT calculations, which allowed us to study the reactant and product evolution corroborated by theoretical simulations. In order to compare the interactions of HBr and HCl with the catalysts, individual pulses of either HBr or HCl molecules were applied to the rutile catalysts (Fig. 3). When comparing the normalised peak responses of HCl and HBr, we observe that the HBr peaks are significantly delayed by 0.44 s (RuO_2_), 0.82 s (TiO_2_), and 0.17 s (IrO_2_), compared to the HCl peaks. This indicates that HBr interacts more strongly with the surface than HCl, which is in line with the HRTEM and DFT results, and the calculated dissociative adsorption energy for RuO_2_(110), which is –2.33 eV for HBr compared to –2.09 eV for HCl on the clean surface. Comparing the catalysts, the order of the HBr peak maxima is as follows: RuO_2_ < IrO_2_ < TiO_2_ at 523, 573, and 623 K (Fig. S9†). The peak position is affected by changing the temperature, and the peak is significantly delayed at higher temperature. This indicates a sizable activation barrier for the overall process during the TAP experiment, which is likely due to underlying surface/subsurface processes (*vide supra*). Furthermore, a difference between the HBr pulses for individual HBr and simultaneous HBr + O_2_ pulsing is observed for IrO_2_ (0.31 s), and TiO_2_ (0.67 s) (Fig. 3). The shift could be due to the reaction of HX and O_2_ to form X_2_ and water, in which the reactants are consumed (Fig. 3 and S10†), leading to a reduction of the integrated peak area. This leads to a shift of the HX peak maximum to shorter times and less tailing of the signal in comparison to the individual pulsed reactant signal, assuming that the pulse size of the reactant was about the same in both individual and simultaneous pulsing experiments.4d,^18^ For RuO_2_, a difference in tailing is observed, but no clear shift of the peak position is visible, which might indicate that the interaction between RuO_2_ and HBr is not significantly affected by the simultaneously pulsed oxygen. For HCl oxidation, only RuO_2_ exhibits a clearly narrower tailing of the HCl response when comparing individual HCl and simultaneous HCl + O_2_ pulses, indicating that only RuO_2_ is significantly active under TAP conditions (Fig. S10†). Thus, only RuO_2_ is considered for further comparative evaluation of HBr and HCl oxidation. A comparison of the O_2_ pulses after individual O_2_ and simultaneous HBr + O_2_ pulsing does not show a clear difference, because the integrated peak area is generally 10 times higher (Fig. S10†). This further indicates the presence of state-defining conditions, as the oxidic surface seems to be unchanged upon exposure to the large O_2_ pulses.15*d* In addition, the halogen surface coverage can be assumed to be very low (*i.e.* <1%), due to the small size of the HX gas pulses compared to the total surface area of the loaded catalysts under ultra-high vacuum (UHV) conditions. Thus, extensive surface or subsurface halogenation of the catalysts during the TAP experiment does not occur.

**Fig. 4:**
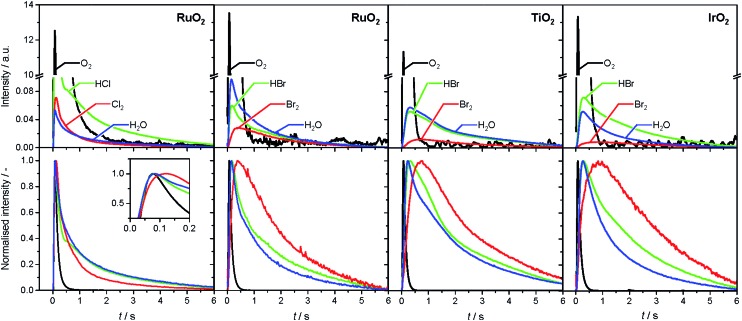
Transient (top) and normalised transient (bottom) responses of reactants (HCl, HBr, O_2_) and products (Br_2_, Cl_2_, H_2_O) at 623 K upon simultaneous pulsing of HX and O_2_ over rutile-type catalysts. The colour code applies to both the top and bottom rows. The break in the *y*-axis in the top plots enables a better visualization of the product responses: Cl_2_, Br_2_, and H_2_O. The inset in the bottom left plot magnifies the region at the maxima of the normalised transient responses.

The product responses of Br_2_, Cl_2_, and H_2_O after simultaneous pulsing of O_2_ with either HCl or HBr are depicted in Fig. 4. When comparing the product responses, it is evident that Cl_2_ production is greater than Br_2_ production over RuO_2_ at the same temperature, which could be explained by the very low detection efficiency of the mass spectrometer for Br_2_ molecules, due to the lower bond strength of Br_2_ (193 kJ mol^–1^) in comparison with Cl_2_ (243 kJ mol^–1^), preventing a direct comparison of their signals. The bond strengths of HBr (363 kJ mol^–1^) and HCl (428 kJ mol^–1^) are comparably high, so no effect on the detection efficiency is assumed. The direct calibration of the quadrupole mass spectrometer (QMS) using pulses of bromine and chlorine was considered, but was not conducted due to the unjustifiable damage expected to occur due to corrosion. However, comparing the signals due to water, which forms in stoichiometric amounts with either Br_2_ or Cl_2_, indicates that the rate of bromine formation must be higher than the rate of chlorine formation. The pulse response size of the water and bromine signals increases in the order: IrO_2_ < TiO_2_ < RuO_2_, indicating the order of activity (Fig. 4, top row). The normalised signals show that the water response always reaches its peak maximum before the halogen response, suggesting that the halogen evolution is slower and can be considered the most energy-demanding step (Fig. 4, bottom row). However, the differences in mass between the water and halogen molecules could effect the observed shift of the peak response in the molecular diffusion regime, hampering in particular a comparison of the water and chlorine responses, due to their small difference in mass. In the case of HBr oxidation, the shift between the HBr and Br_2_ peak maxima can be safely interpreted as a measure of the activity of the catalysts. The shifts decrease in the order IrO_2_ (0.60 s) > TiO_2_ (0.43 s) > RuO_2_ (0.22 s), which is the opposite order to that observed for product formation.

The faster bromine evolution observed over TiO_2_ compared to IrO_2_ is a crucial piece of experimental evidence that reaffirms the computational results. This proof could not be obtained through atmospheric pressure steady-state experiments because the apparent activity of both oxides in HBr oxidation was very similar. This is likely due to a compensation effect similar to that which occurs in the Deacon reaction on RuO_2_ at high coverage.5*a* The compensation that occurs between both materials is proposed to result from a breaking of the scaling relationships, as discussed recently.^7^ In the present work, the pulsing experiments in TAP allow the elementary steps of the reaction network to be decoupled. In this sense, it is possible to investigate only the desorption process, as it occurs under very low coverage and state-defining conditions, as explained above. Thus, in the absence of high bromine coverage we were able to observe the intrinsic differences in bromine evolution between TiO_2_ and IrO_2_, as postulated in ref. 7.

In order to verify the reaction mechanism of HBr and HCl oxidation, pump-probe experiments were conducted, in which either the HX or the O_2_ pulse was delayed by a distinct time period (Fig. S11 and S12†). In the O_2_ (pump) and HX (probe) pulsing experiment, product formation can be observed after either pulse. The amount of product formed after the HX probe pulse is much larger, which is due to the available oxygen on the surface following the pump pulse (Fig. S11†). This is analogous to the HX (pump) and O_2_ (probe) pulsing, in which the product formation after the HX pump pulse is also significant. The large amount of product formed after the O_2_ pulse can be related to oxygen-assisted product evolution (Fig. S12†), as described for HCl oxidation in recent studies.5*a*

**Fig. 5:**
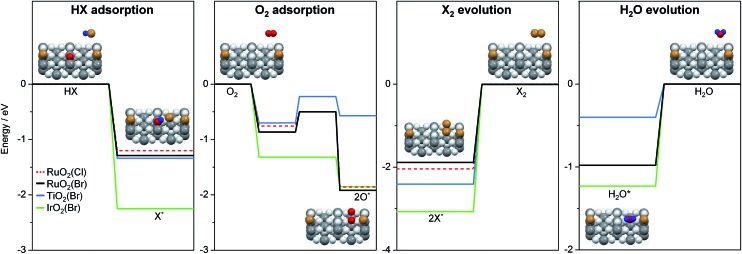
Key steps of reactant adsorption and product evolution steps for HBr (solid lines) and HCl (dashed line) oxidation on halogenated rutile-type surfaces. The H_2_O evolution steps over RuO_2_(X) for HBr (black) and HCl (red) oxidation overlap. The full reaction profile is shown in Fig. S13.† The insets illustrate the reaction steps on a rutile surface. Colour code: metal atoms (dark grey), Br (brown), H (blue), and O (light grey and red). O atoms are coloured in red if they participate in the reaction.

To further interpret our experiments and to complement earlier results,^7^ we computed the reaction profiles for HBr and HCl oxidation over halogenated rutile surfaces (Fig. S13†). For better comparison, the most important reaction steps are depicted in Fig. 5. The basic surface sites can strip a proton from HX, resulting in a surface hydroxyl and an adsorbed halide ion. Re-oxidation was identified as the key step in Deacon catalysis on RuO_2_ to close the cycle; this is the step where most differences appear between RuO_2_ and TiO_2_. O_2_ adsorption on metallic surfaces like RuO_2_ and IrO_2_ is limited only by the number of free active sites available on the surface.^19^ On TiO_2_, oxygen chemistry is necessarily related to defect chemistry.^7^ In the present case, the defect chemistry originates from one of the reactants (HBr), so it can be partially understood as auto-catalytic. This activity is only possible for HBr, as the Cl (3p) levels in Cl are far too low in energy compared to the Br (4p) levels in Br (resulting in a stronger adsorption for Cl), and thus the energy required to eliminate Cl_2_ from the surface is far too high for the reaction to occur in the temperature window for TiO_2_ activity.^7^ In addition, for HX oxidation to take place, sites capable of both acid–base and redox interactions are required.^7^ Metal oxides with metallic properties exhibit both of these characteristics to a reasonable degree, but the redox properties are more subtle for semiconductors, in which defect levels play the leading role. In view of the product evolution, Cl_2_ recombination is more energy-demanding than Br_2_ recombination, with the recombination energy of the latter over different surfaces increasing in the following order: RuO_2_(Br) (1.9 eV) < TiO_2_(Br) (2.44 eV) < IrO_2_(Br) (3.05 eV).¶
¶For TiO_2_, two different configurations were tested and the most stable one is taken into account.
^,^^20^ This coincides with the product evolution observed in the simultaneous pulsing experiments. The recombination of two halogen atoms on the surface is the most energy-demanding reaction step in all investigated systems. Water evolution is less energy-demanding by more than 1 eV (Fig. 5). For the latter, the hydroxyl groups can recombine over the surface, generating an adsorbed water molecule that can then leave the surface. These water-related steps have very similar mechanisms on all of the surfaces.

### Impact of the halogen surface coverage

**Fig. 6:**
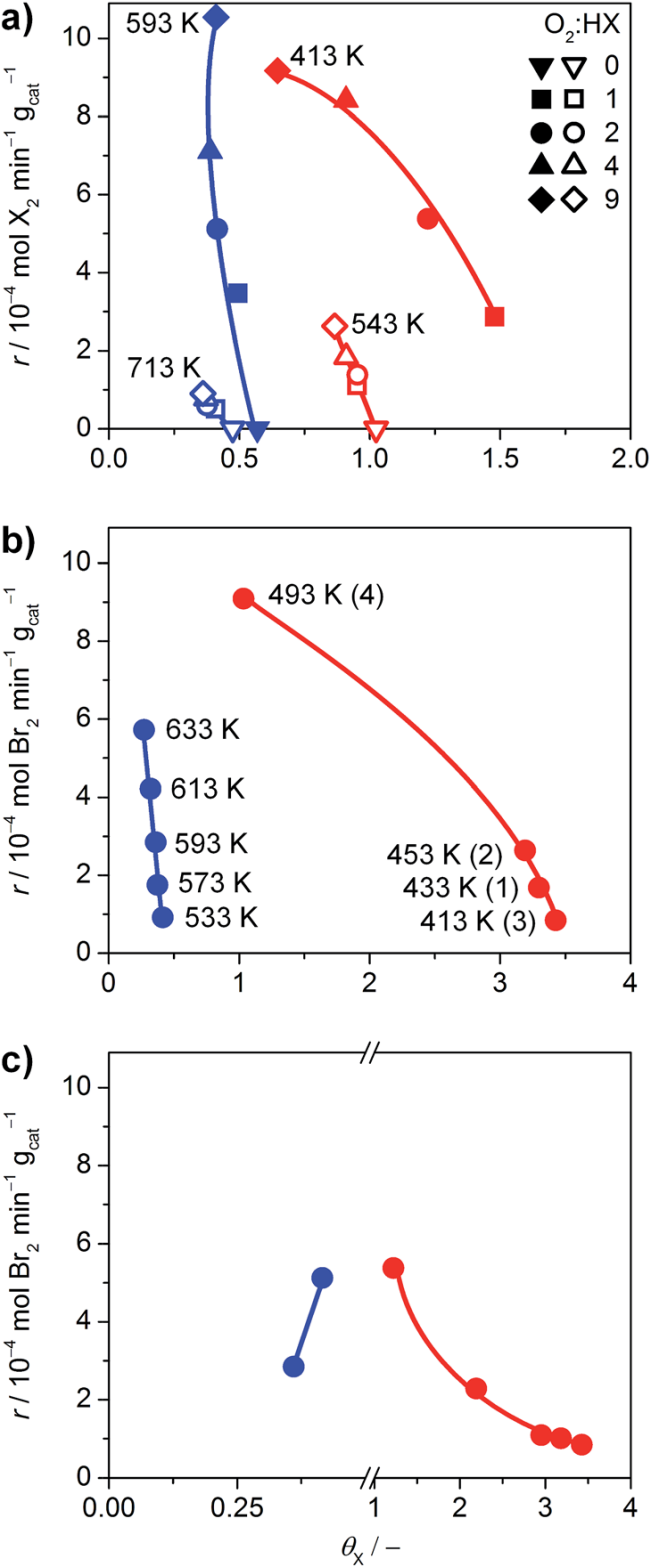
(a) Rate of halogen formation of HBr (solid symbols) and HCl (open symbols) oxidation as a function of the halogen coverage over TiO_2_ (blue) and RuO_2_ (red), showing the dependence on the O_2_ : HX feed ratio at constant temperature. The O_2_ : HX ratio was increased from 0 to 9, except for the HBr oxidation over RuO_2_, where the order was inverted from 9 to 1. (b) Dependence of the rate on the catalyst bed temperature at O_2_ : HBr = 2. The temperature for TiO_2_ was increased from 533 K to 633 K. The order of measurements for RuO_2_ is indicated by the numbers in parentheses. (c) Rate of bromine formation *versus* the bromine coverage. The data points (O_2_ : HBr = 2, 593 K for TiO_2_, and 413 K for RuO_2_) were taken from the measurements shown in (a and b), and Fig. S14.† Coverages exceeding unity indicate the occurrence of subsurface halogenation.

The halogen coverage on the catalysts was determined by *operando* PGAA under real-life conditions for RuO_2_ and TiO_2_. An investigation of IrO_2_ was unfortunately not possible, because the neutron excitation and radioactive decay of Ir leads to the emission of very intense gamma radiation that rapidly saturates the detector, and the high baseline created by the Ir matrix overshadows the Br signal.^21^ Thus, the comparison focused on TiO_2_ and RuO_2_ at variable O_2_ : HX feed ratios and temperatures (Fig. 6), where the HCl and HBr conversion reached maximum values of 29% and 84%, respectively.

As shown in Fig. 6a, TiO_2_ exhibits halogen coverages of 0.57 and 0.48 for Br and Cl respectively, at O_2_ : HX = 0. With increasing oxygen partial pressure, these values decrease to 0.4 (Br) and 0.36 (Cl). The Cl coverage on RuO_2_ is close to 1 at O_2_ : HCl = 0 and reaches 0.86 at O_2_ : HCl = 9, which is in line with recent studies.^5^ However, in agreement with our HRTEM and DFT results, RuO_2_ exhibits subsurface bromination when varying the O_2_ : HBr ratio from 0 to 9, reaching a degree of bromination that exceeds the theoretical full surface coverage by 300% (Fig. S14†). Therefore, the tests on RuO_2_ in HBr were conducted under slightly different conditions, with the O_2_ : HBr ratio decreasing from 9 to 1, whereby the Br coverage increased from 0.65 to 1.48 (Fig. 6a). The dependence of the halogen coverage on the variation of the bed temperature, as indicated in the respective plots, is very similar to the dependence of the halogen coverage on the oxygen partial pressure (Fig. 6b and S15†). On increasing the temperature, the halogen coverage on TiO_2_ drops from 0.41 (Br) and 0.54 (Cl) to 0.27 (Br) and 0.38 (Cl), respectively. The halogen coverages on TiO_2_ are significantly lower compared to RuO_2_, which is in agreement with the *ab initio* thermodynamics. In Fig. 6c, data points from the different experiments are combined to show the effect of bromine coverage on the rate under the same reaction conditions. The bromine coverage on TiO_2_ is so low that a slight increase in surface bromine content leads to a higher activity. On the other hand, the bromine coverage on RuO_2_ is above the theoretical surface coverage, and further increases are detrimental to the activity of RuO_2_ in HBr oxidation (Fig. 6c). Thus, an optimal halogen coverage between the observed values for TiO_2_ and RuO_2_, at which the bromine formation rate would be maximal is likely to exist.

**Fig. 7:**
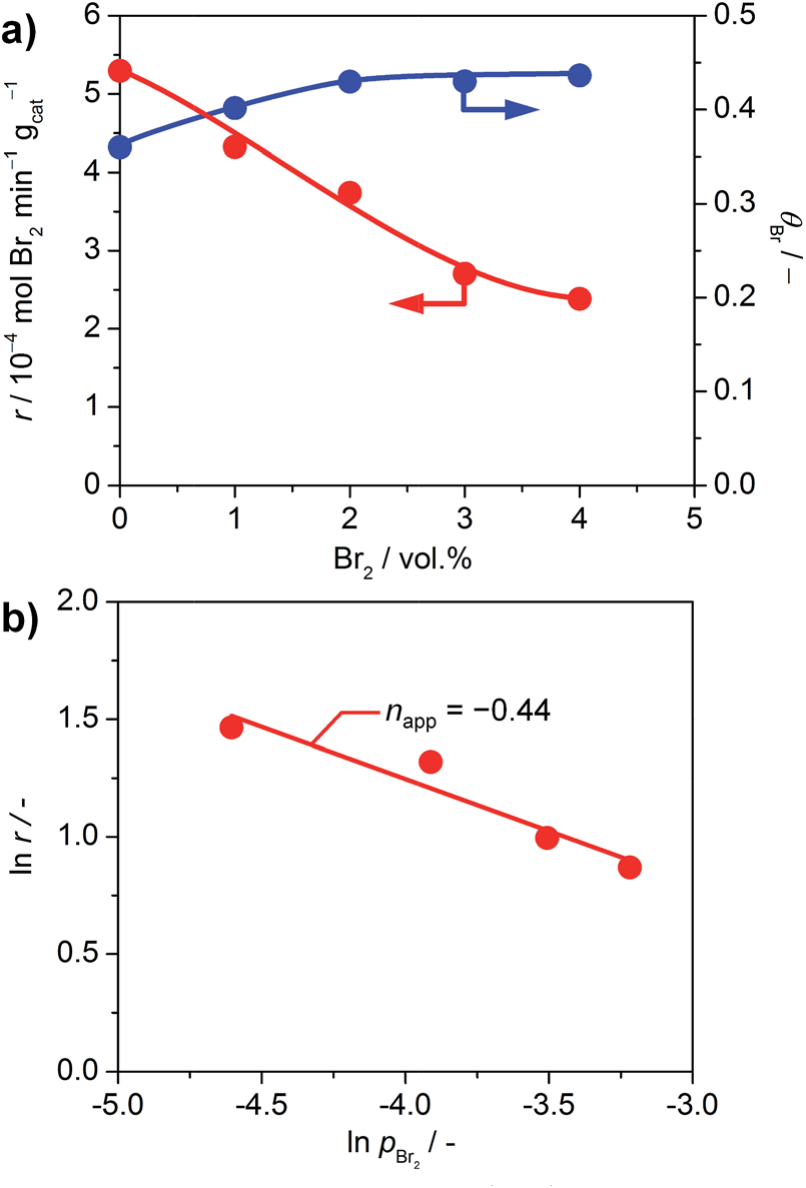
(a) Rate of bromine formation (red) and bromine surface coverage (blue) *versus* the volumetric feed of bromine over TiO_2_ at 593 K and O_2_ : HBr = 2. (b) Rate of bromine formation *versus* partial pressure of bromine.

The impact of product inhibition was investigated for TiO_2_ by adding bromine to the gas feed (Fig. 7a). The addition of 4 vol% Br_2_ causes a distinct decrease in the bromine formation rate of 55%. However, the bromine coverage stabilises at 0.43 after increasing the bromine feed to 2 vol%. This indicates that there is no significant bromination of the catalysts through the addition of bromine gas, which is in agreement with the endothermic adsorption of Br_2_ on clean TiO_2_ as calculated by DFT (0.69 eV with respect to ½Br_2_); a bifunctional adsorption as proposed by Li and Metiu^20^ does not improve the adsorption energy. Recent studies highlighted that the gas-phase thermodynamic equilibrium of HBr oxidation is shifted far to the side of the products.6*d* Thus, the decreased activity is not linked to a shift in the thermodynamic equilibrium, but is due to the reaction kinetics. The determination of the apparent reaction order gives a value of –0.44 for the impact of bromine on the reaction rate (Fig. 7b). This value is close to the theoretical apparent order of –0.5, which describes the dissociative adsorption of bromine on the active site. Hence, it seems plausible that a competitive adsorption between HBr and Br_2_ occurs on the small number of active sites of TiO_2_ and likely depends on the defect states left on its surface, originating from doping by the small amount of Br taken up by the surface. As a comparison, the inhibition effect of Cl_2_ on RuO_2_ in the Deacon reaction is –1.00.5*a* The larger inhibitions induced for RuO_2_ catalysts are due to the fact that site competition is favoured on metallic substrates, compared to semiconductors like TiO_2_. The ultimate reason for this is that once doped, the Ti centres are no longer chemically equivalent to each other, and thus they discriminate between the adsorption of electron-rich and electron-poor fragments. In comparison, the conduction band in metals screens out these effects, and it is not possible to differentiate the chemical nature of the cations.

### Halogen chemistry on rutile surfaces

With all the data described in the previous sections, a generalised picture of the chemistry of halogens on rutile catalysts emerges. Although the chemical nature of HCl and HBr might appear to be virtually identical at a first glance, their interactions with the set of potential catalysts are markedly different. Initially, the surface basicity and the acidic character of HX leads to an exothermic adsorption pattern for all combinations. But, depending on the particular oxide/hydrogen halide pair, the adsorption can lead to: (i) a minor fraction of oxygen lattice replacement (TiO_2_/HBr), (ii) a medium level of oxygen lattice replacement and a large surface coverage (RuO_2_/HCl), or (iii) an extensive surface modification with concomitant subsurface occupation and the growth of a second phase on the surface (RuO_2_/HBr). The factors controlling these differences are related to: (i) the nature of the oxides and (ii) their limited ability to store extra electrons in defect states (TiO_2_), while for the metallic oxides (RuO_2_ and IrO_2_), the extent of halide replacement is mostly governed by the relative energies of the M–O and M–X bonds.

The activity of the rutile is then completely governed by the halide content in the material; to quote Paracelsus, the dose makes the poison. Thus, the mechanism might range from only involving surface positions (Langmuir–Hinshelwood-type mechanism) as for HBr on TiO_2_, through a continuum that involves the activation and/or replacement of surface lattice oxygen atoms (*i.e.* closer to a Mars–van-Krevelen-type mechanism). The active catalyst must then be obtained through control of the oxygen pressure in the gas-phase, which can limit the halide content to only surface positions, thus rendering the catalyst active. The single exception to this behaviour is the semiconductor material TiO_2_. For TiO_2_, the self-doping occurring as a result of replacement of the surface oxygen atoms by halide is self-limited, due to the energy cost of accumulating electrons in surface and subsurface states.^22^ The activity is then linked to the presence of forbidden energy areas, from which it is possible to add/extract electrons, and how effective the alignment of the energy levels is.

## Conclusions

We have investigated HBr and HCl oxidation on rutile RuO_2_, IrO_2_, and TiO_2_ catalysts by applying an integrated approach consisting of advanced methods to analyse and compare their surface chemistry. We found that the structural stability of the HX oxidation catalysts depends on the interactions of the hydrogen halide molecules with the catalyst surface. The combined HRTEM, DFT, and TAP studies reveal that the amorphisation of the RuO_2_ particle surface after treatment with HBr is due to structural distortion of the clean surface geometry after extensive bromination. Notice, however, that extensive bromination and thus surface amorphisation also occurred in HBr oxidation. The impact of HCl is much more limited, leading to less pronounced distortion as a result of the comparably unfavourable energetics of oxygen substitution, restricting Cl replacement to the surface. The computed bromine substitution into the TiO_2_ lattice is endothermic, suggesting only limited potential for bromination. The TAP experiments support these results, indicating a stronger interaction of rutile-type materials with HBr than with HCl. Further pulsing experiments in TAP indicate that halogen evolution can be considered to be the most energy-demanding step, with the activity of the catalysts for HBr oxidation increasing in the following order: IrO_2_ < TiO_2_ < RuO_2_. This coincides with the computational results. Both HBr and HCl oxidation reactions follow a Langmuir–Hinshelwood-type mechanism on surfaces with a low to moderate degree of halogenation, as confirmed through pump-probe experiments. The halogen uptake is the key descriptor that links stability and activity of the catalytic systems. On RuO_2_, the steady-state surface halogen coverage reaches 86–100% for chlorine and 75–300% for bromine, proving that subsurface bromination occurs, agreeing with the HRTEM and DFT studies. A decrease in the halogen coverage on RuO_2_ can be induced by increasing the operation temperature and elevating the O_2_ : HX feed ratio. Still, our tools cannot describe in detail the mechanism for surface/subsurface bromination. It is possible that some degree of subsurface Br uptake takes place early in the reaction process, before the surface is completely saturated with bromine. This will affect the catalyst activity in the long term, even at higher temperatures. In contrast, the halogen coverage of TiO_2_ is limited to 30–50% as a result of its self-doping mechanism with bromine. An increase in the temperature and the O_2_ : HX feed ratio induces higher activity, but does not significantly affect the halogen coverage on TiO_2_. The addition of Br_2_ to the gas feed over TiO_2_ does not affect the bromine coverage, but inhibits the reaction at an apparent order of –0.44, indicating a competitive adsorption of Br_2_ and HBr on the active sites. As a result of this study, we consider it highly desirable to combine the exceptional low-temperature activity of RuO_2_ with the stability of TiO_2_ against extensive surface halogenation by designing new Ru–Ti mixed oxide catalysts for HBr oxidation with an optimal balance between activity and stability.

## Supplementary Material

Supplementary information
