# Supplementary material for: Interplay between surface chemistry and performance of rutile-type catalysts for halogen production
Source: Chem Sci. 2016 Jan 27;7(5):2996–3005. doi: 10.1039/c5sc04247j (PMC6004776; doi:10.1039/c5sc04247j)
Supplement: Supplementary file 1 [file SC-007-C5SC04247J-s001.pdf]

Electronic Supplementary Material (ESI) for Chemical Science.
This journal is © The Royal Society of Chemistry 2016

Electronic Supplementary Information (ESI)

Interplay between surface chemistry and performance on rutile-type catalysts for halogen production

Maximilian Moser,[a] Vladimir Paunović,[a] Zhen Guo,[a] Laszlo Szentmiklósi,[b] Miguel G. Hevia,[c] Michael Higham,[c] Núria López,*[,c] Detre Teschner,*[,d] and Javier Pérez-Ramírez*[,a]

[a] Institute for Chemical and Bioengineering, Department of Chemistry and Applied Biosciences, ETH Zurich, Vladimir-Prelog-Weg 1, 8093 Zurich, Switzerland.

[b] Centre for Energy Research, Hungarian Academy of Sciences, Konkoly-Thege Miklós street 29-33, 1121 Budapest, Hungary.

[c] Institute of Chemical Research of Catalonia, ICIQ, Barcelona Institute of Science and Technology, BIST, Av. Països Catalans 16, 43007 Tarragona, Spain.

[d] Fritz-Haber-Institute of the Max Planck Society, Faradayweg 4-6, 14195 Berlin, Germany.

*Corresponding authors. E-mails: nlopez@iciq.es (N.L.)
teschner@fhi.mpg.de (D.T.)
jpr@chem.ethz.ch (J.P.-R.)

Additional Computational Details

DFT calculations were performed over 33 different systems (besides the clean $RuO_2(110)$ surface) containing up to 6 Br atoms per $p(2\times1)$ surface unit cell of the $RuO_2(110)$ slab. For these calculations, the functional of choice was PBE-D2, thus including dispersion contributions. These may be relevant for the very polarisable Br atoms (S. Grimme, *J. Comp. Chem.,* 2006, **27**, 1787). Various configurations were tested for different relative positions of Br atoms (Fig. S3). For each configuration, the energy for the replacement of O atoms with Br was calculated using the reference reaction $RuO_2 + 2n\text{HBr} \rightarrow RuO_2@Br_n + nH_2O + n/2Br_2$, where n is the number of Br atoms incorporated. The most energetically favourable configurations were obtained for each Br incorporation. *Ab initio* thermodynamics were performed using these lowest energy configurations to determine the number of halogen atoms incorporated into the unit cell under typical experimental conditions as a function of the partial pressures p(HBr), $p(H_2O)$, and $p(Br_2)$ (Figs. 2, S6, and S7). An analogous procedure was followed to determine the extent of Cl uptake of $RuO_2(110)$ and the outcome is presented in the same figures.

Similar calculations were also performed to investigate the Br uptake of $TiO_2(110)$. However, they are less exhaustive since the extent of bromination of titanium oxide is much lower compared than that of ruthenium oxide. Thus, DFT calculations were performed for 4 distinct configurations investigating the incorporation of up to 2 Br atoms into a $p(2\times2)$ cell at 6-coordinate bridging sites in comparison to the clean slab (Fig. S8). As for RuO_2 halogenation, the structures were relaxed and replacement energies calculated. The lowest energy configurations were used in *ab initio* thermodynamics studies of the surface composition as a function of p(HBr) and $p(H_2O)$ (Figs. 2 and S6).

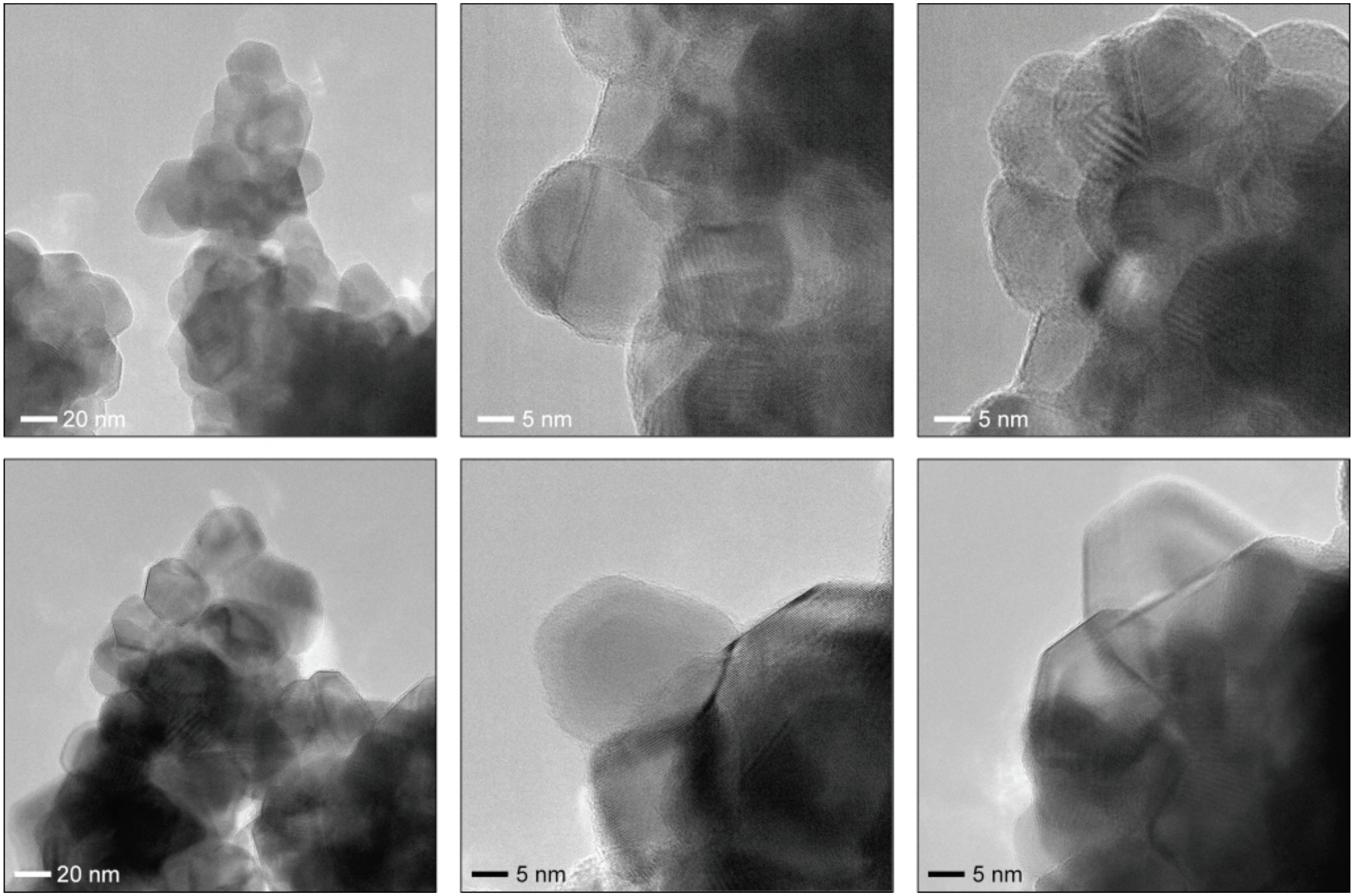

Fig. S1. HRTEM of RuO_2 treated in HBr at 393 K (top micrographs) and HCl at 543 K (bottom micrographs) for 3 h.

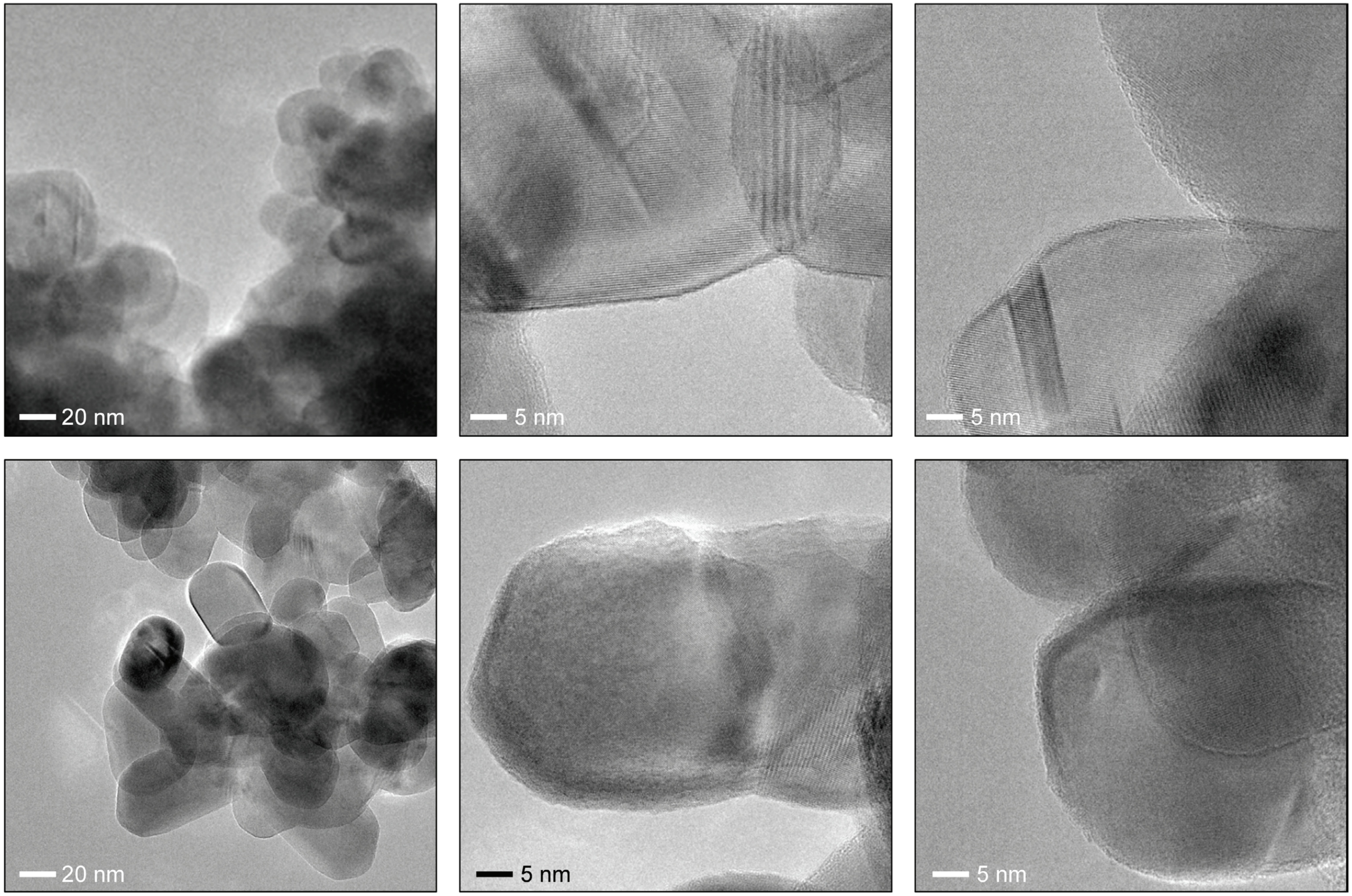

Fig. S2. HRTEM of TiO_2 treated in HBr at 573 K (top micrographs) and HCl at 633 K (bottom micrographs) for 3 h.

a)

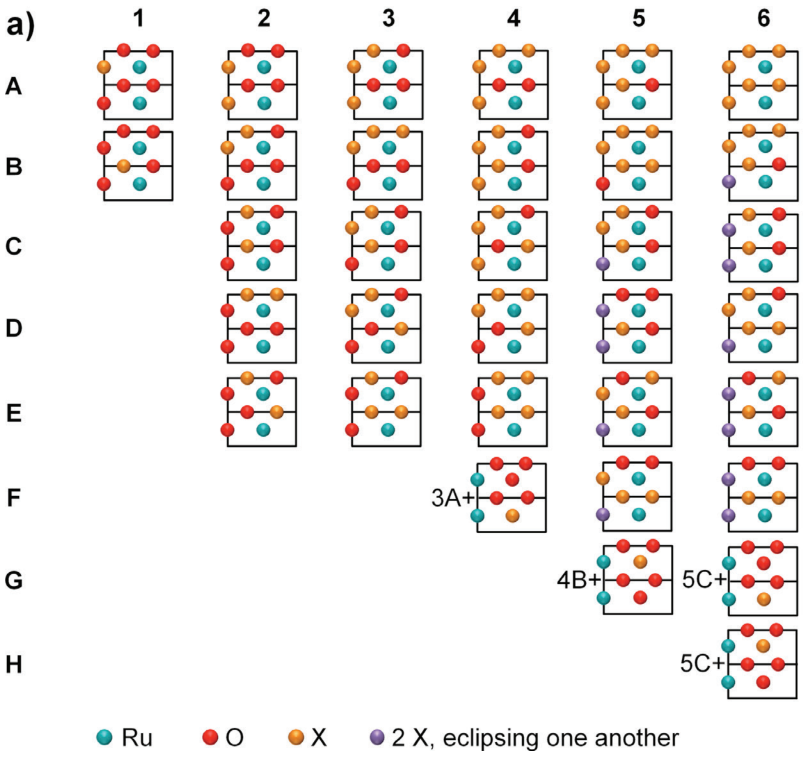

b)

Free energy of halide atom substitution / eV						
	1	2	3	4	5	6
A	-1.462<br>-0.817	-1.973<br>-0.926	-1.520<br>0.137	0.849<br>3.428	0.476<br>3.122	6.423<br>9.322
B	0.334<br>1.071	-0.928<br>0.363	1.563<br>3.245	-1.373<br>0.712	5.422<br>8.781	1.296<br>5.050
C		0.700<br>1.834	-0.589<br>1.122	0.576<br>2.622	-0.503<br>1.831	3.244<br>8.661
D		2.614<br>3.558	0.344<br>2.308	0.911<br>3.157	2.076<br>6.498	1.296<br>5.050
E		1.257<br>2.784	1.991<br>3.629	7.512<br>9.015	1.438<br>3.461	2.625<br>7.827
F				0.198	1.744<br>2.954	6.504<br>10.136
G					0.210<br>2.316	0.647<br>3.515
H						0.303<br>3.207

Fig. S3. Schemes of the incorporation of halogen atoms (X = Br, Cl) into a (2×1) cell for the RuO_2(110) surface (a). The number of substituted halide atoms (1-6) as well as their relative position in the lattice (A-H) was computed for 33 different configurations. Configurations 4F, 5G, 6G, and 6H depict the halogen substitution into the subsurface layer, whereby the surface configurations are identical to 3A, 4B, and 5C, as indicated in (a). The free energy values of Br (blue values) and Cl (red values) atom substitution for all configurations are listed in (b). Blue boxes indicate the lowest free energy cases.

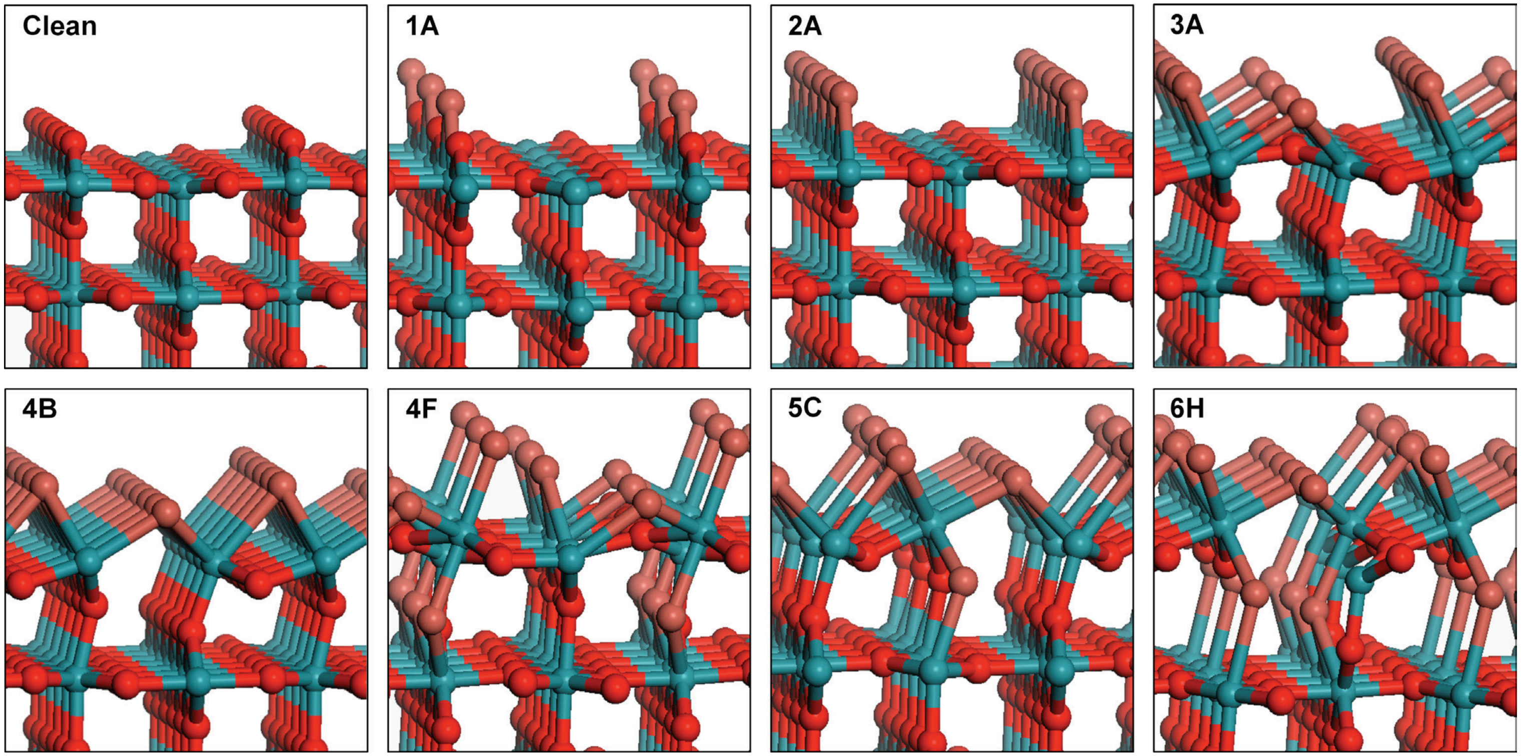

Fig. S4. Clean and Br substituted RuO_2 surfaces having the lowest free energies as listed in Figs. 2 and S3b. Colour code: Ru (blue), O (red), and Br (brown). Notice the distorted rutile structure upon a certain degree of bromination.

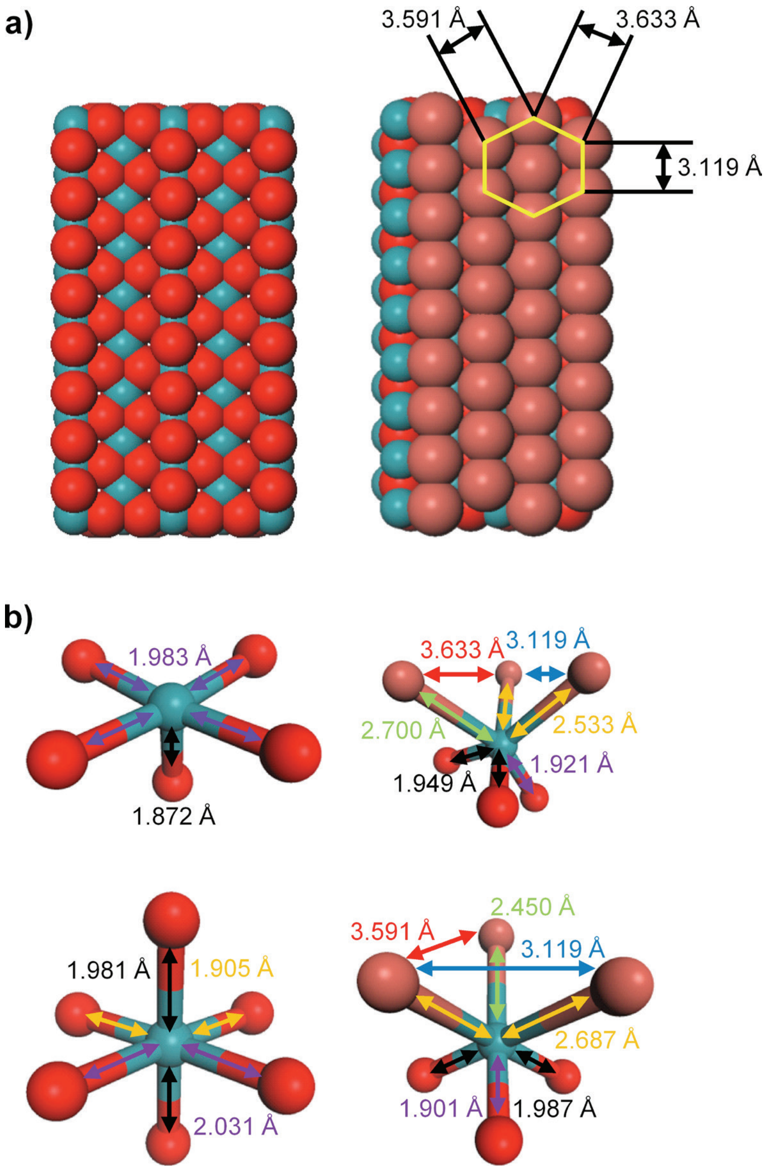

Fig. S5. Regular RuO_2(110) slab (left) and 4B configuration (right) are presented for comparison depicting the distinctive hexagonal arrangement of bromine atoms on the surface (a). Local surface coordination environments of the Ru_{cus} site (top) and 6-coordinate Ru site (bottom) for the regular slab (left) and for the 4B configuration (right) are shown in (b). Colour code: Ru (blue), O (red), and Br (brown).

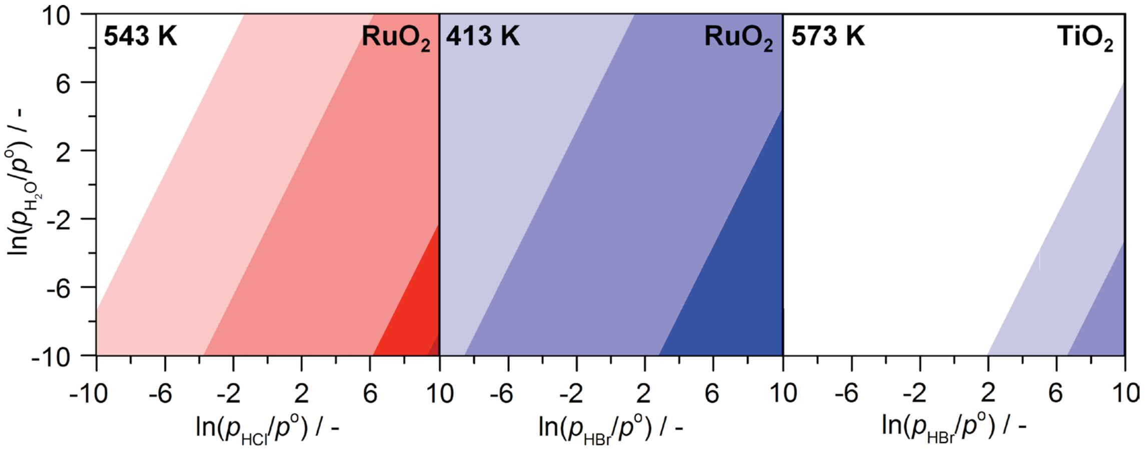

Surface	Energy / eV	Surface	Energy / eV	Surface	Energy / eV
6H	3.207	4B	-1.373	2B	1.328
4B	0.712	2B	-0.928	1A	0.431
2A	-0.926	1A	-1.462	Clean	0
1A	-0.817				
Clean	0				

Fig. S6. *Ab initio* thermodynamics of the Cl (red) and Br (blue) substitution on MO_2(110) surfaces. The colour shades denote the surface configurations in Figs. S3 and S8 having the lowest energies under the simulated conditions. The partial pressures of Cl_2 and Br_2 were fixed at 1 bar.

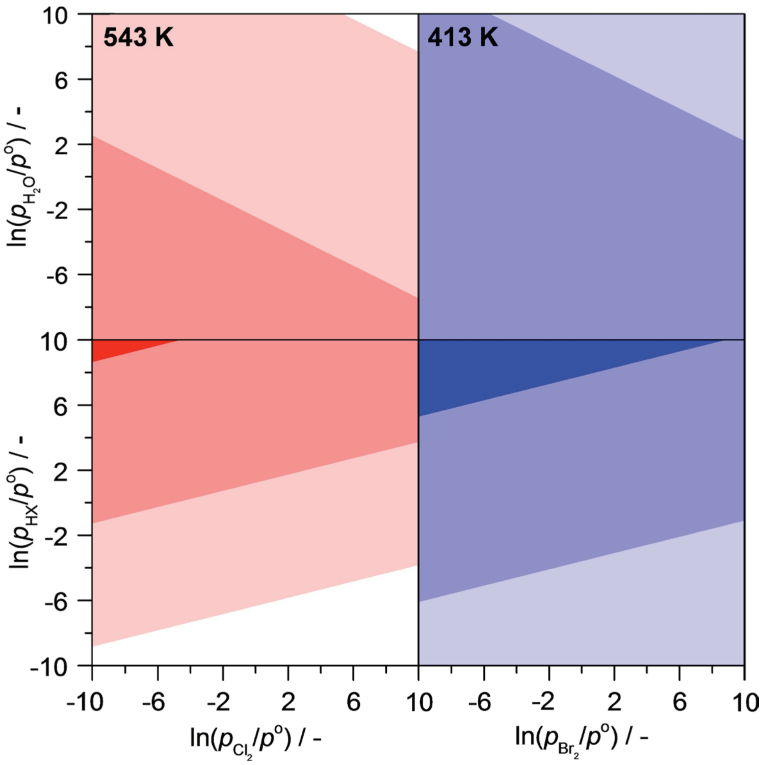

Surface	Energy / eV	Surface	Energy / eV
4B	0.712	4B	-1.373
2A	-0.926	2B	-0.928
1A	-0.817	1A	-1.462
Clean	0		

Fig. S7. *Ab initio* thermodynamics of the Cl (red) and Br (blue) substitution on RuO_2(110) surfaces. The colour shades correspond to the surface configurations in Fig. S3 having the lowest energy under the simulated conditions. The partial pressures of HX (top) and H_2O (bottom) were fixed at 1 bar.

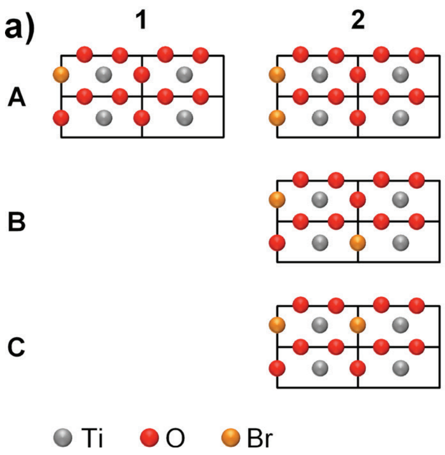

b)

	Free energy of halide atom substitution / eV	
	1	2
A	0.431	1.589
B		1.328
C		1.344

Fig. S8. Schemes of the incorporation of bromine atoms into a (2×2) cell for the TiO_2(110) surface (a). The number of substituted halide atoms (1-2) as well as their relative position in the lattice (A-C) was computed for 4 different configurations. The free energy values of Br substitution for all configurations are listed in (b). Blue boxes indicate the lowest free energy cases.

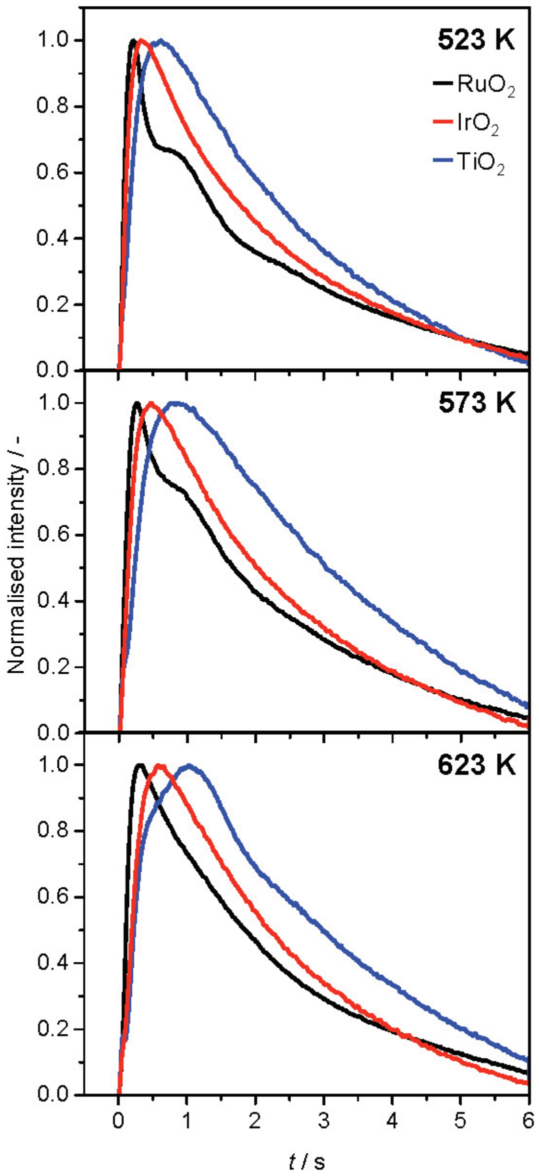

Fig. S9. Normalised transient responses of HBr upon individual pulsing of HBr over rutile-type catalysts at different temperatures.

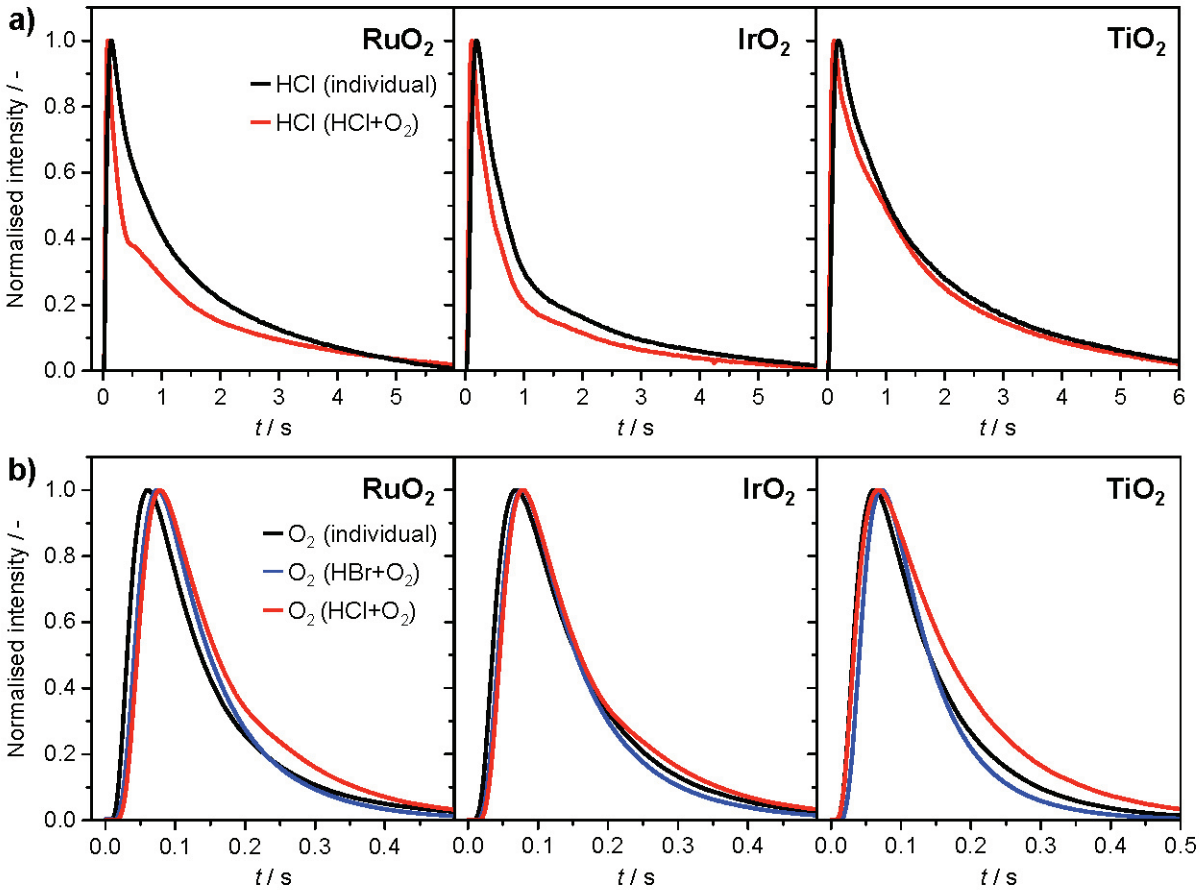

Fig. S10. Normalised transient responses of HCl at 623 K upon HCl (black) and HCl+O_2 (red) pulsing (a). Normalised transient responses of O_2 at 623 K upon O_2 (black), HBr+O_2 (blue), and HCl+O_2 (red) pulsing (b).

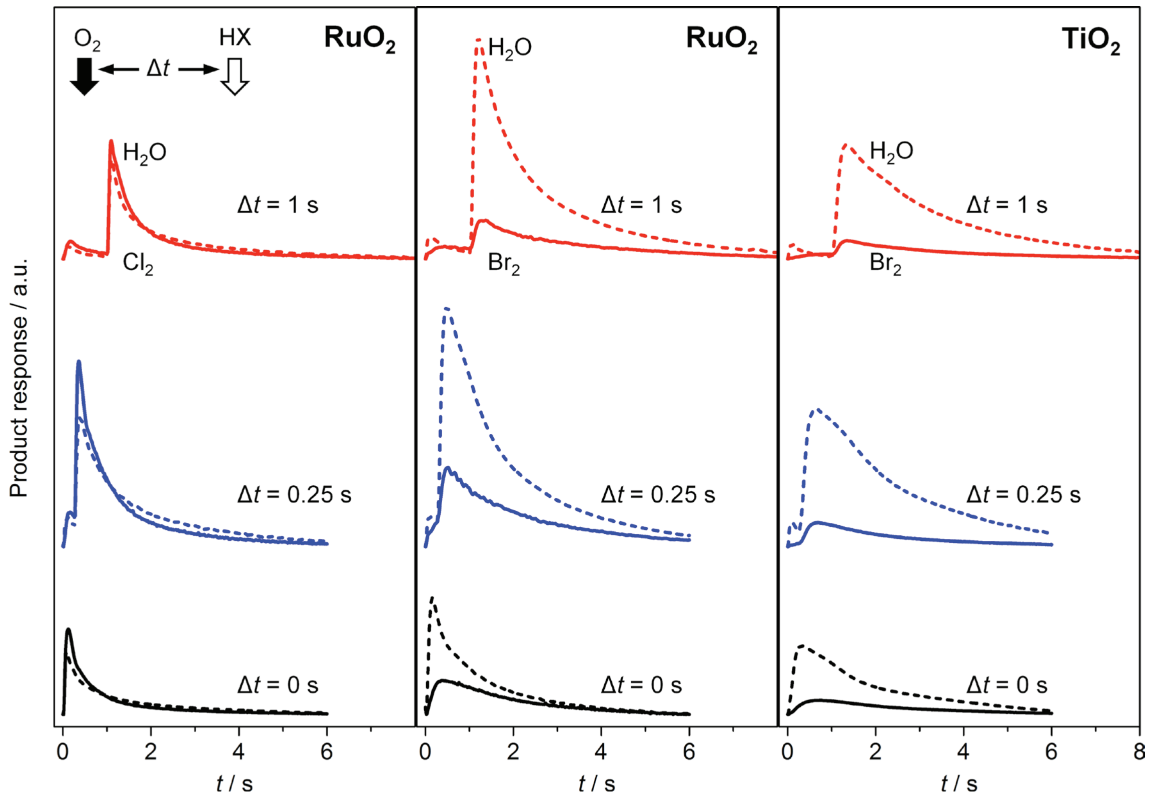

Fig. S11. Transient responses of Br_2 and Cl_2 (solid lines) as well as H_2O (dashed line) in pump-probe experiments with O_2-HBr and O_2-HCl at variable time delay (Δt) over RuO_2 and TiO_2 at 623 K. The order of the pump and probe molecules is depicted in the top-left corner.

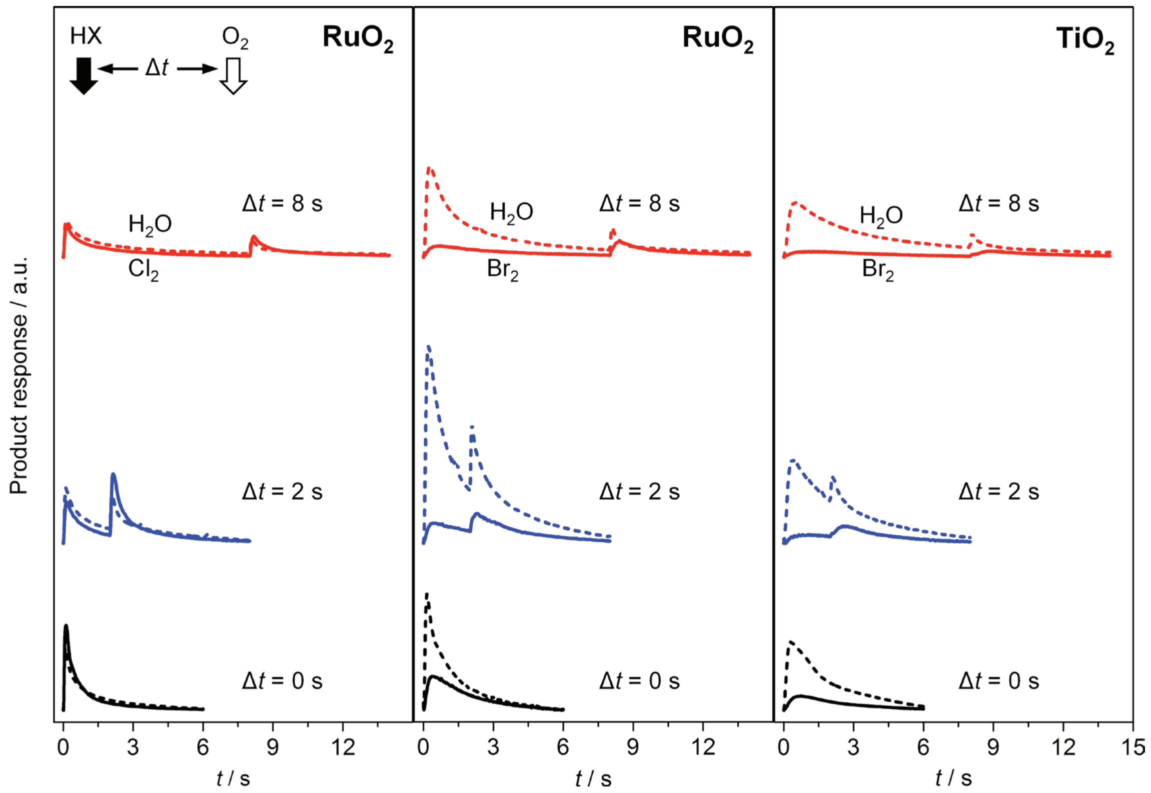

Fig. S12. Transient responses of Br_2 and Cl_2 (solid lines) as well as and H_2O (dashed line) in pump-probe experiments with HBr-O_2 and HCl-O_2 at variable time delay (Δt) over RuO_2 and TiO_2 at 623 K. The order of the pump and probe molecules is depicted in the top-left corner.

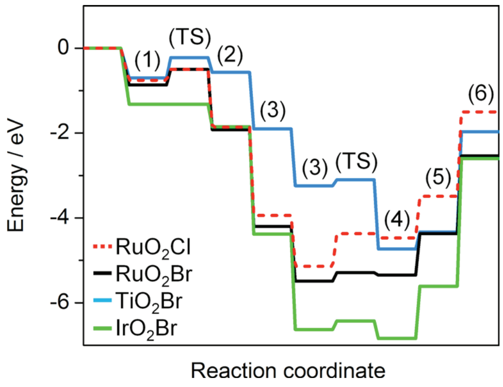

Fig. S13. Reaction profiles of HBr (solid lines) and HCl (dashed line) oxidation on halogenated rutile-type catalysts. Herein, (1) is the molecular O_2 adsorption and (2) is the dissociated state after the transition state (TS). Subsequently, two HX molecules are adsorbed in steps (3), followed by the transition state (TS) for the OH recombination to water (4). Finally, both water (5) and molecular halogen (6) are desorbed from the surfaces. The profile corresponds to half a stoichiometric reaction: $O_2 + 2HX \rightarrow O^* + H_2O + X_2$, thus step (3) occurs only twice and an oxygen atom is left on the surface in the final state.

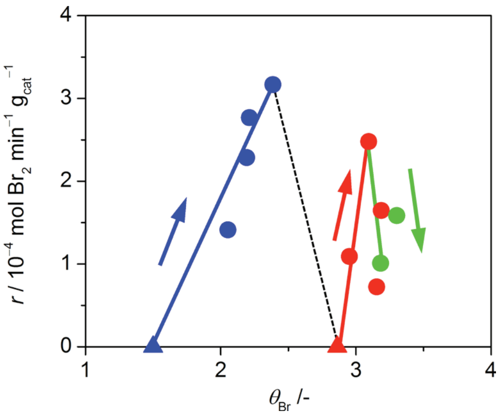

Fig. S14. Rate of bromine formation versus bromine coverage on RuO_2. After pre-treatment at O_2:HBr = 0 and 393 K (blue triangle), the temperature was gradually increased from 393 K to 433 K in steps of 10 K (blue circles). Afterwards, the pre-treatment was repeated (red triangle) before increasing the O_2:HBr ratio in the order 1, 2, 4, and 9 at 413 K (red circles). Finally, the O_2:HBr ratio was decreased from 9 to 4 and 2 at 413 K (green circles). Despite the increase in temperature and O_2 content of the feed mixture, RuO_2 gradually increases its bromine uptake, which exceeds the maximal theoretical coverage by up to 3 times and thus indicates subsurface bromination of RuO_2.

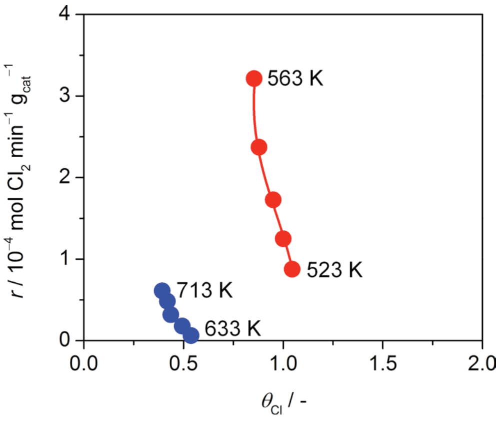

Fig. S15. Rate of chlorine formation versus chlorine coverage over TiO_2 (blue circles) and RuO_2 (red circles) at O_2:HCl = 2 and different temperatures. The temperature was increased from 633 K to 713 K in steps of 20 K for TiO_2, and from 523 K to 563 K in steps of 10 K for RuO_2.
